# ERP correlates of unexpected word forms in a picture–word study of infants and adults

**DOI:** 10.1016/j.dcn.2012.01.003

**Published:** 2012-01-16

**Authors:** M.D. Duta, S.J. Styles, K. Plunkett

**Affiliations:** Department of Experimental Psychology, University of Oxford, South Park Rd, Oxford OX1 3UD, UK

**Keywords:** Event related potentials (ERP), Vowel perception, Mispronunciation detection, Speech perception, Language development

## Abstract

We tested 14-month-olds and adults in an event-related potentials (ERPs) study in which pictures of familiar objects generated expectations about upcoming word forms. Expected word forms labelled the picture (word condition), while unexpected word forms mismatched by either a small deviation in word medial vowel height (mispronunciation condition) or a large deviation from the onset of the first speech segment (pseudoword condition).

Both infants and adults showed sensitivity to both types of unexpected word form. Adults showed a chain of discrete effects: positivity over the *N*_1_ wave, negativity over the *P*_2_ wave (PMN effect) and negativity over the *N*_2_ wave (*N*400 effect). Infants showed a similar pattern, including a robust effect similar to the adult *P*_2_ effect. These observations were underpinned by a novel visualisation method which shows the dynamics of the ERP within bands of the scalp over time. The results demonstrate shared processing mechanisms across development, as even subtle deviations from expected word forms were indexed in both age groups by a reduction in the amplitude of characteristic waves in the early auditory evoked potential.

## Graphical abstract

**Graphical abstract:**

**Figure:**
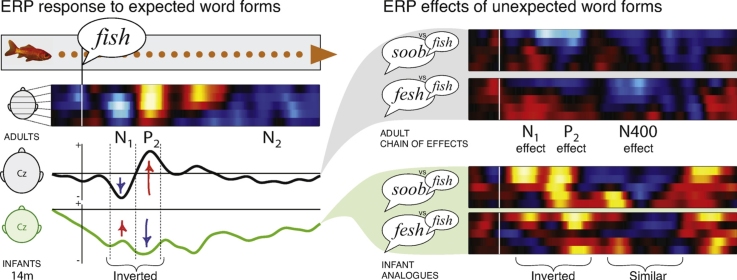

**Highlights:**

* Adults and infants show similar effects generated by unexpected word forms. * 14-Month-olds show an analogue of the adult PMN effect. * Infants detect mispronounced vowels from 225 ms, and larger changes 75 ms earlier. * Adult semantic *N*400 effects are discrete from expectation-related PMN effects. * Novel data visualisation method integrates spatial and temporal information.

## 1 Introduction

To understand natural speech, adults require detailed perception of individual speech sounds, well-defined lexical representations of word forms, and the ability to access these representations rapidly as incoming speech is processed. During the first year of life, infants demonstrate finely grained perception of individual speech sounds (see Kuhl, 2004, for a review) including discrimination between subtly different vowels (see Polka and Bohn, 2003). However, when word learning emerges towards the end of the first year of life, it is unclear how infants’ perceptual abilities are integrated into their developing representations of word forms. In particular, little evidence exists about the representation of vowels embedded in word forms in the early lexicon, or about the neural correlates of vowel identification. What is needed is a more refined understanding of the neural correlates of segmental integration early in lexical development. The temporal sensitivity of event related potentials (ERPs) makes them ideal for investigations of this kind.

Infants in their second year are known to respond to deviations from the standard pronunciation of known words, detecting mispronunciations of both consonants and vowels (Bailey and Plunkett, 2002, Ballem and Plunkett, 2005, Mani and Plunkett, 2007, Mani et al., 2008, Swingley, 2005, Swingley and Aslin, 2000, Swingley and Aslin, 2002). Their segmental knowledge is detailed, exhibiting graded sensitivity to deviation (White and Morgan, 2008, Mani and Plunkett, 2011), even for newly learnt words (Mani and Plunkett, 2008, Yoshida et al., 2009).

Furthermore, access to segmental information is very fast, and can be employed even before a whole word has been heard. For example, in picture–word matching tasks, infants are able to extract sufficient information from the first 200 ms of a word to reliably shift their gaze away from pictures which do not match the unfolding speech stream (Swingley et al., 1999, Fernald et al., 2001). The speed of this online, incremental processing of speech makes it likely that the neural correlates of segmental integration will be evident in the early auditory evoked potential.

In both adults and infants, auditory processing reaches cortical regions shortly after audio onset: within 10 ms and 20 ms, respectively (Wilkinson and Jiang, 2006). Early cortical auditory evoked potentials in adults have a characteristic morphology known as the *PNP* complex, made up of a succession of three waveform deflections: *P*_1_, a positive wave peaking at 50 ms; *N*_1_, negative, 100 ms; *P*_2_, negative, 180–200 ms. This complex is followed by a longer-lasting negativity, the *N*_2_ wave. The *PNP* complex is evident from birth, and undergoes maturational changes during the first years of life (for reviews see Crowley and Colrain, 2004, Wunderlich and Cone-Wesson, 2006).

The physical properties of sounds, and the contexts in which they occur generate a chain of effects modulating the adult auditory evoked potential. The *N*_1_ wave is modulated by acoustic features including intensity and frequency, (Näätänen and Picton, 1987). The *P*_2_ wave is modulated by expectations about upcoming speech—an effect reported in both auditory-only priming paradigms (Newman and Connolly, 2009), and in cross-modal picture-label paradigms (Desroches et al., 2008). This short-lasting relative negativity is also known as a phonological mapping negativity (PMN), and has been described as the earliest effect generated by a mismatch from expected speech sounds (see Steinhauer and Connolly, 2008, ch. 9). The *N*_2_ wave is modulated by the well-documented long-lasting *N*400 effect, where relative negativity is understood to index effortful semantic integration (see Kutas and Federmeier, 2011, for a review).

Despite a certain degree of temporal overlap, the PMN and the *N*400 are thought to be discrete effects, as has been demonstrated by Desroches et al. (2008), who manipulated phonological and semantic mismatches independently. In response to phonological onset mismatches, they report a short-lasting negative deflection during the *P*_2_ wave (230–310 ms). In response to semantic mismatches occurring without phonological onset mismatch, they found longer-lasting enhanced negativity in a later time window (300–600 ms).

Infant auditory evoked potentials have also been reported to be modulated by differences in the contextual relevance of word forms (i.e., mismatching, or completely novel words) in picture contexts (Friedrich and Friederici, 2005, Mani et al., 2011), and by the familiarity of auditory word forms (Mills et al., 2004, Mills et al., 2005, Thierry et al., 2003, Vihman et al., 2007). There is consistency across audio-only paradigms, in which responses to rare words exhibit reduced amplitude for the negative peak at around 200 ms (11 months-of-age, peaks within the 170–248 ms time window, Thierry et al., 2003; 10 and 11 months-of-age, but not 12 months-of-age, peaks within the 180–310 ms time window, Vihman et al., 2007).

Taking the findings from auditory only paradigms together, it becomes clear that unexpected word forms generate in adults more negativity over the positive *P*_2_ wave, and in infants more positivity over an early negative wave. A striking similarity is apparent between age groups if it is noted that both effects represent a reduction in the amplitude of a wave in the early evoked potentials.

However, in picture–word mismatch paradigms, the pattern of reported infant effects is less consistent. Mani et al. (2011) report that in 14-month-olds pseudowords and medial vowel mispronunciations elicited short-lasting left lateralised negativity over a 200–300 ms time window (an effect with the opposite polarity to infant effects in audio-only paradigms), followed by longer-lasting negativity in the 400–600 ms time window (an effect with the same polarity as adult *N*400 effects). In a study of infants and adults, Friedrich and Friederici (2005) report the characteristic pattern of effects in adults, with pseudowords eliciting a short-lasting (100–250 ms) relative positivity over the *N*_1_ wave, followed by a short-lasting (250–400 ms) relative negativity over the *P*_2_ wave, then a long-lasting negativity over the *N*_2_ wave (windows over 400 to 1200 ms). However, the 12-month-old infants exhibited a long-lasting effect, with pseudowords generating more positivity over windows from 100 to 500 ms, while for the 19-month-olds, there was evidence for an early frontally distributed positivity (windows covering 100–500 ms) overlapping with the beginnings of a more adult-like central negativity starting at 400 ms.

In this context, it is unclear whether the effects elicited in response to pseudowords and vowel mispronunciations in Mani et al. (2011) constitute a different type of segmental processing effect from those reported in Friedrich and Friederici (2005). Closer comparison between the two studies is complicated by the lack of common stimulus manipulations across common age groups. Thus it is unclear how infant mispronunciation effects relate to the more widely documented modulations of the adult auditory evoked potentials in response to auditory word forms. Better understanding of segmental processing in infancy can only be achieved through direct comparisons between infants’ and adults’ responses to the same stimuli.

The current study explores modulations of the ERP generated by word form expectations created by picture contexts, comparing two age groups, at two extremes of linguistic development: experienced adult language users, and 14-month-old infants at the beginning of their word learning journey. Auditory stimuli either matched the expected word form, or mismatched it in one of two ways. In the pseudoword condition auditory stimuli mismatched from the onset of the first speech segment. In the mispronunciation condition auditory stimuli mismatched in the height of the word-medial vowel.

For adults we predict a chain of effects generated by mismatches from the expected word form: short-lasting sub-lexical mismatch effects reflected over the *PNP* complex, followed by longer-lasting *N*400 effect indexing semantic integration. Note that, for adults, each mispronunciation could represent an unfamiliar articulation, for example a novel dialect, while pseudowords will not act as labels for the pictures. Hence, the effects induced by the two mismatching conditions may have different profiles.

For 14-month-old infants we predict that expectations about upcoming word forms will generate short-lasting sub-lexical mismatch effects. However, infants at this stage of lexical development may not reject unfamiliar word forms (pseudowords or mispronunciations) as plausible labels for familiar objects. Therefore, it is unclear whether they will show coherent adult-like *N*400 semantic integration effects.

We present a novel data visualisation method which shows the dynamics of the evoked potentials within bands of scalp over time. This visualisation enables refined study of chains of discrete effects with spatial and temporal overlap, and permits an informative comparison between the two age groups. Comparisons between the two age groups provide critical evidence about shared processing mechanisms.

## 2 Materials and methods

### 2.1 Participants

We report results from 19 monolingual native English speaking adults with a mean age of 21 years (SD= 3.1), and 18 infants from monolingual English speaking families with a mean age of 14.1 months (SD= 0.2). Participants were included in this sample if they provided a minimum of 12 artifact-free trials per condition.

The adult participants were volunteers recruited mainly from the university student community and were given course credit or paid £10 for their participation. Infants were recruited from a pool of families who had previously expressed interest in participating in developmental studies, and were offered a gift for their participation. The study was approved by the Central University Research Ethics Committee.

### 2.2 Experimental design

**Fig. 1:**
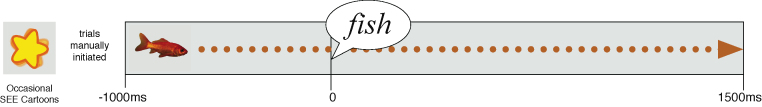
Trial timeline.

This study involved recording the electroencephalogram (EEG) while participants attended to a series of pictures of familiar objects and animals in an auditory labelling context. Each trial consisted of the presentation of a picture on screen for 2500 ms, during which a token of speech was presented with a fixed stimulus onset asynchrony (SOA) of 1000 ms (see Fig. 1). To maintain adults’ attention to both visual and auditory stimuli, a monitoring task was included at the end of each trial. The task required responses to on-screen questions about the category of the picture, or the onset consonant of the audio token.

Test words were short words containing a lax vowel in a stressed, word medial position. Each word was paired with a mispronunciation which differed in the height of the medial vowel, generating pairs which differed by either high-mid or mid-low contrasts. The mispronunciations did not yield existing English words. Only words familiar to 14-month-old infants were used in this study (min. comprehension rate of 40%, MacArthur–Bates CDI: Dale and Fenson, 1996), resulting in 12 word-mispronunciation pairs, along with four additional monosyllabic words, selected to be paired with ‘filler’ items, which were always named correctly and were excluded from analyses.

For the pseudoword condition, 12 monosyllabic pseudowords were chosen from the ARC Nonword Database (Rastle et al., 2002). Words were paired with pseudowords in which the onset consonant differed in place of articulation, and the vowel, in height and backness. A full list of stimulus items is given in the supplementary material (S1) along with detailed information about stimulus pitch (S2).

Each participant saw each picture twice in each of the three auditory conditions, for a total of six presentations, making up 72 trials per individual. For example, a picture of a fish appeared twice with the word *fish*, twice with the mispronunciation *fesh, and with two different pseudowords, e.g., *dom and *soob. Variation in the pseudoword pairings prevented novel word learning during the testing session. Each presentation of a picture was separated by at least seven trials. Pictures were presented in a pseudo-randomised order, with no picture occurring in the same condition more than once per half of the experimental session.

### 2.3 Stimuli

Audio stimuli were produced in citation form by a female native speaker of British English with a standard Southern accent. Stimuli were recorded in a single session in a sound-attenuating booth using a solid state recorder sampling at 44.1 Hz, in 16 bit stereo. Stimuli were filtered to remove hiss and hum, and edited to remove head and tail clicks using Goldwave 5.23. Auditory stimuli had a mean duration of 695 ms (SD = 112 ms).

Visual stimuli were 16 high-resolution colour photographs (1024×768 pixels) depicting typical exemplars of each of the test and filler words. The photographs were digitally edited to remove backgrounds, adjust colour, and remove distracting features (e.g., clothing labels).

### 2.4 Equipment

Visual stimuli were presented centrally on a LCD monitor on a 5% grey background. The screen was 38 cm × 30 cm, creating a viewing angle of approximately 26° from side to side. Audio stimuli were presented via two speakers centrally located above the screen. Stimuli were presented using Presentation (version 13.0.01.23.09, Neurobehavioral Systems, Inc.). Parallel port gamepads were used for stimulus triggering, online trial marking, and adult responses.

The EEG was recorded from 21 locations on the scalp using Compumedics Quik-Caps with Ag/AgCl sintered electrodes arranged according to the International 10–20 system of electrode placement (American Electroencephalographic Society, 1994). For adult participants, the vertical and horizontal electrooculogram (EOG) were recorded with electrodes above and below the left eye (VEOGU and VEOGL), and electrodes placed laterally to the external canthi of the left and right eye (HEOGL and HEOGR). For the infant participants, the VEOGL was measured with an electrode placed below the left eye, VEOGU was approximated by the signal recorded from Fp1, while HEOGL and HEOGR were approximated using the signals recorded from F7 and F8.

The EEG and EOG signals were acquired with Neuroscan Scan 4.3 via NuAmps sampling at 1000 Hz. During recording, the signals were referenced to the left mastoid and band-pass filtered for 0.1–100 Hz. At the start of each recording session, impedances of all electrodes were below 5 kΩ.

Artifact rejection and data processing were conducted offline in MATLAB (Version 7.7.0.471, R2008b, The MathWorks, Inc., Natick, MA) using EEGLab (Delorme and Makeig, 2004), and custom routines. Statistical analysis was performed with SPSS Statistics 17.0.1.

### 2.5 Experimental procedure

Adult and infant participants were tested in a specially designed recording booth, where monitor and speakers were built into a plain grey wall, and no distractions were visible. Adult participants, and caregivers holding infant participants, were seated in a centrally positioned chair.

Adult participants were instructed to try to minimise blinks as well as body and eye movements while the pictures were on the screen, and to respond to the monitoring task quickly and accurately. They used controls on the right-hand side of their gamepad to answer the yes/no question, and to trigger the next trial when they were ready to proceed. In the infant study, an experimenter monitored the behaviour of the infant from the recording booth, triggered trials when the infant attended the screen, and marked unattended trials for exclusion from analysis, using the hand-held gamepad. The testing session ended when infants ceased to attend to the screen, or became very active.

To maintain participants’ attention, trials were manually interspersed with a series of 30 custom animations: the Soothers, Engagers and Eye-catchers (SEE, see supplementary material S3 for details). The inclusion of manual trial initiation and SEE cartoons greatly increased the number of attended trials per recording session, compared with two infant pilot groups.

## 3 Data processing and visualisation

### 3.1 Data processing

The EEG signals were re-referenced to the average of the left and right mastoid channels. Custom zero-phase filters were applied (minimum-order Butterworth filters, high-pass: 3 dB attenuation at 1 Hz, 20 dB attenuation/octave; low-pass: 3 dB attenuation at 15 Hz, 40 dB attenuation at 50 Hz) to remove muscle and drift artifacts. The filtered EEG was segmented into epochs containing a 200 ms baseline preceding audio onset, and 800 ms following audio onset (1000 ms in total). For each epoch, signals from each channel were baseline corrected to the median of the signal over the baseline period. Fillers and unattended trials were excluded.

Finally, to detect and remove epochs corrupted by extreme values and ocular artifacts, custom Matlab routines automatically screened each epoch. Epochs were excluded if the signal from any electrode exceeded a threshold for maximum absolute actual value (adult: 50 μV, infant: 100 μV), or if the signal from the mastoid sites exceeded a threshold for maximum dynamic range (adult: 25μV, infant: 50 μV). Ocular horizontal and vertical artifacts were detected by testing bipolar vertical EOG (VEOGL − VEOGU) and bipolar horizontal EOG (HEOGL − HEOGR) (adult: 35 μV, infant: 75 μV). Following these exclusions, all participants included in analyses gave a minimum of 12 attended, artefact free epochs per condition.

### 3.2 Data visualisation

In the presentation of ERP data, two commonly accepted methods are (a) grand average ERP waveforms at each electrode site, and (b) static topographic maps showing scalp distribution of the mean amplitude of the grand average ERP over a specific time window (see Picton et al., 2000). The first method shows dynamic change in the signal, but the discrete nature of each plot makes it difficult to monitor patterns emerging over groups of adjacent electrodes. By contrast, topographic maps interpolate data between electrodes to provide visualisation of patterns across the scalp, but these ‘slices of frozen time’ can mask the dynamic temporal structure of events. In response to these limitations, we introduce a third visualisation method, which integrates temporal and spatial information into a single plot. Data is pooled across bands of electrodes as a means of collapsing one dimension of the scalp, allowing visualisation of three variables on a two dimensional plane: *x* =time, *y* =scalp band, colour = ERP amplitude. These dynamic scalp bands provide a colour-coded representation of the continuous dynamics of scalp regions over time, without restricting the visualisation to a specific time window.

**Fig. 2:**
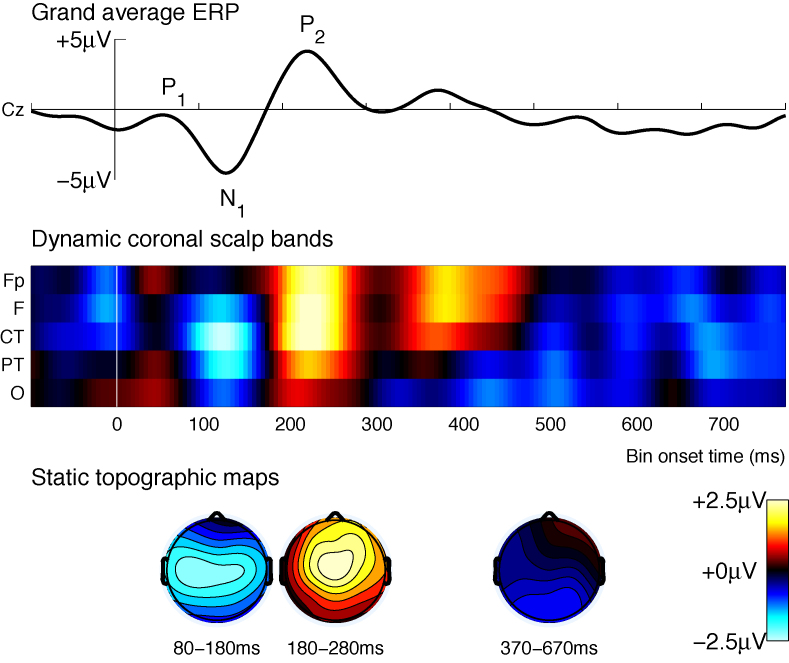
Adult response to expected word forms time-locked to auditory stimulus onset. (Top) Grand average ERP from Cz showing first 800 ms of auditory evoked potentials, following a 100 ms baseline period. (Middle) Dynamic scalp bands showing mean amplitude ERP over 25 ms bins with 5 ms staggered onsets. Data pooled across five scalp bands in coronal orientation: frontal pole Fp=(Fp1, Fp2), frontal F=(F7, F3, Fz, F4, F8), centro-temporal CT=(T3, C3, Cz, C4, T4), parieto-temporal PT=(T5, P3, Pz, P4, T6) and occipital O=(O1, O2). (Bottom) Static topographic maps showing mean amplitude ERP over three time windows: 80–180 ms, 180–280 ms and 370–670 ms.

**Fig. 3:**
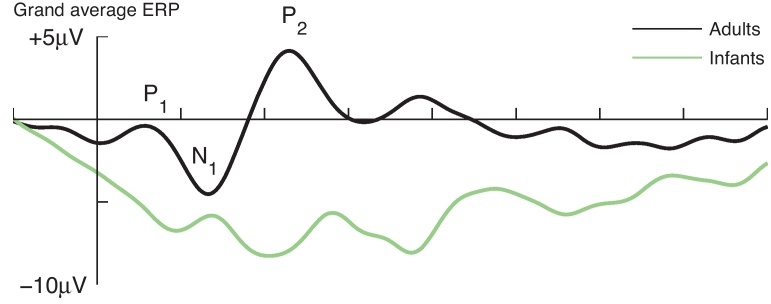
Adult and infant response to expected word forms time-locked to auditory stimulus onset. Grand average ERP from Cz showing first 800 ms of auditory evoked potentials.

Fig. 2 gives all three visualisations of the adult ERP in response to words, where the relationship between the three plots is made clear. Waveforms from a single electrode (Cz) are plotted above dynamic scalp bands oriented to the coronal plane, accompanied by static topographic maps of mean amplitude ERP over three time windows: 80–180 ms, 180–280 ms, and 370–670 ms. The dynamic scalp bands show mean amplitude ERP over 25 ms sliding bins with 5 ms staggered onsets. Within each bin, data was pooled across a series of five scalp bands in coronal orientation from front to back of the scalp: frontal pole (Fp1, Fp2), frontal (F7, F3, Fz, F4, F8), centro-temporal (T3, C3, Cz, C4, T4), parieto-temporal (T5, P3, Pz, P4, T6) and occipital (O1, O1). The combination of perspectives in this figure allows detailed study of subtle morphology and dynamics of the auditory evoked potentials. For comparison between age groups, Fig. 3 presents ERP waveforms from Cz in response to words, for both adults and infants.

**Fig. 4:**
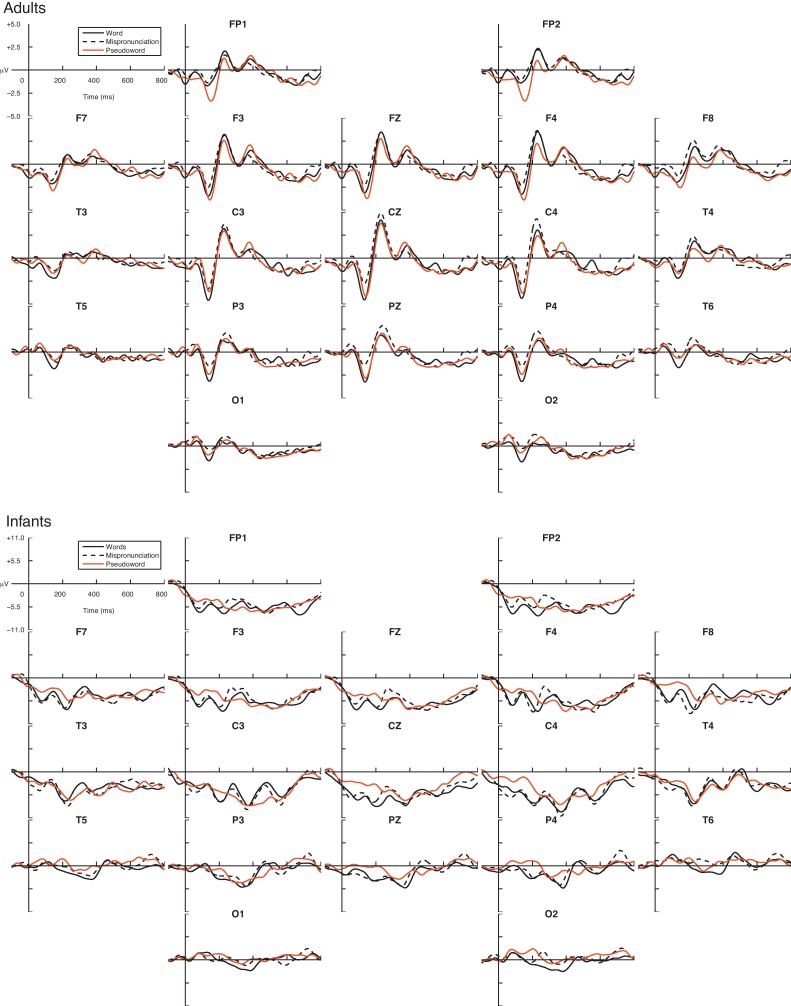
Response to the three conditions time-locked to auditory stimulus onset. Grand average ERP at each electrode sites: (Top) adults and (Bottom) infants.

**Fig. 5:**
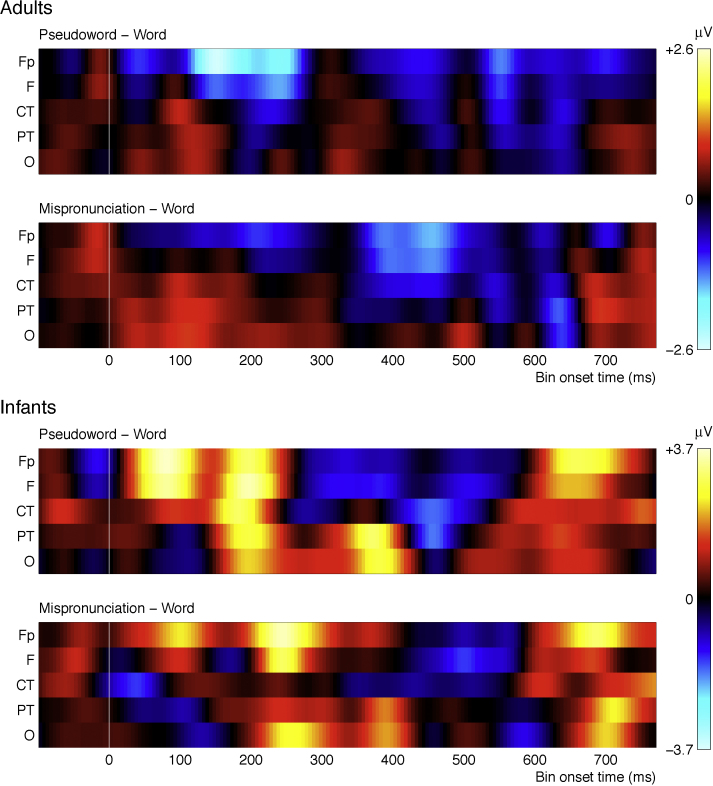
Dynamic coronal scalp bands for grand average ERP mean amplitude differences: mismatching conditions minus word condition: (Top) adults and (Bottom) infants.

**Fig. 6:**
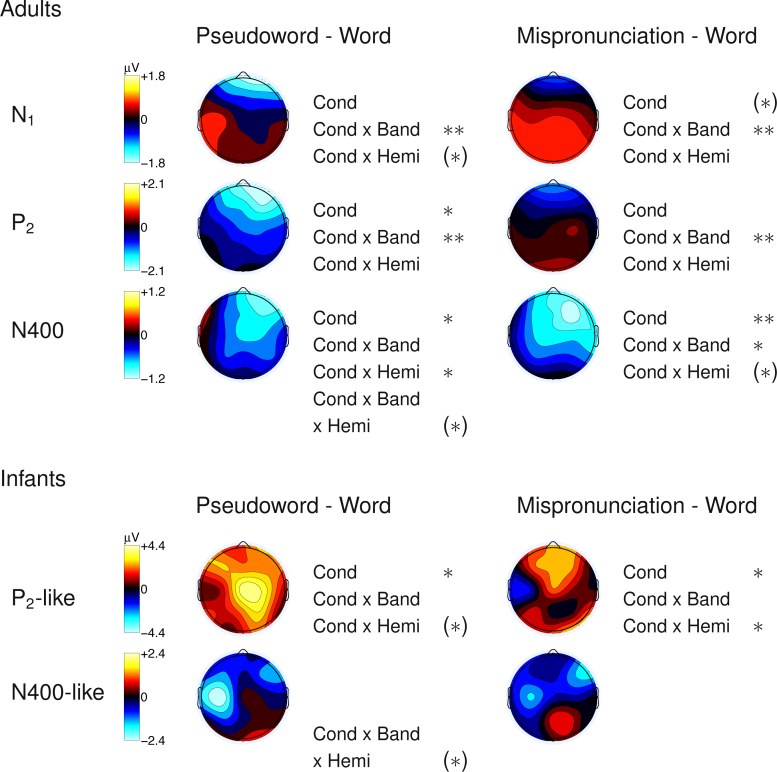
Static topographic maps of mean amplitude ERP difference: mismatching conditions minus word condition. Summary of main effects and interactions of condition. (Top) Adults: *N*_1_ (80–180 ms), *P*_2_ (180–280 ms), *N*400 (370–670 ms). (Bottom) Infants: *P*_2_-like (pseudoword-word: 150–250 ms; mispronunciation-word: 225–325 ms), *N*400-like (400–600 ms). **p < . 01; *p < . 05; (*) *p* < . 07.

Grand average ERP waveforms for all three conditions are given in Fig. 4 for participants in each age group. To enable clarification of the dynamics of effects induced by unexpected word forms, Fig. 5 depicts dynamic coronal scalp bands showing the difference between each unexpected condition and the expected, word condition for each age group. To support interpretation of statistical analyses, Fig. 6 gives static topographic maps of the difference between each mismatching condition and the word condition, in windows of interest.

### 3.3 Data analysis

**Fig. 7:**
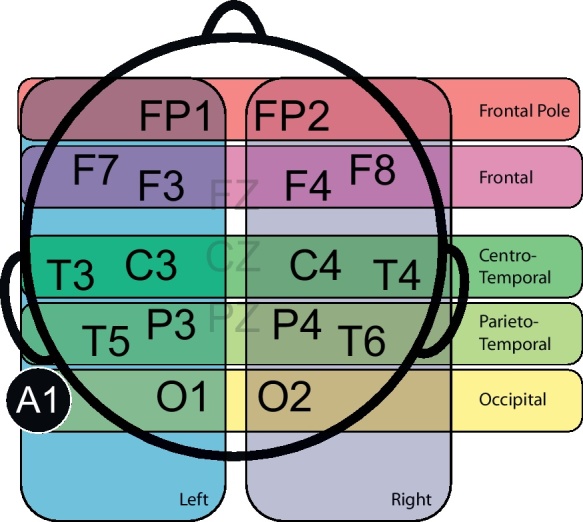
Electrodes included in ANOVA analyses: five bands with coronal orientation, each subdivided by hemisphere.

Statistical analysis was performed with Condition × Band × Hemisphere repeated measures ANOVAs for which the scalp was divided into five bands of electrodes with coronal orientation, each of which was subdivided by hemisphere: frontal pole (Fp1/Fp2), frontal (F7, F3/F4, F8), centro-temporal (T3, C3/C4, T4), parieto-temporal (T5, P3/P4, T6), occipital (O1/O2) (see Fig. 7). The analyses were conducted on mean amplitude ERP values calculated over fixed time windows in which each unexpected word form condition was compared to the expected, word condition.^1^ ANOVAs comparing the two unexpected conditions were also performed and are reported where effects or interactions with condition were significant. Greenhouse–Geisser corrections have been applied where required.

The literature predicts both short-lasting effects over the early stages of the ERP and longer lasting *N*400-effects. In order to identify the precise onsets and offsets of the windows of interest to capture these effects, an objective algorithm based on analysis of variance over consecutive bins was employed (in accordance with Picton et al., 2000, p. 142, I(i)). The algorithm consisted of a series of repeated measures ANOVAs comparing each unexpected word form condition with the expected, word condition using mean amplitude ERP over rolling bins of fixed duration, with onsets staggered every 5 ms. Windows of interest were defined as those bins for which the *F*-value reached a local maximum, and effects or interactions with condition were significant below the *α*-level of 0.5 for adults and 0.10 for infants. To capture short-lasting effects emerging over the *PNP* complex, 100 ms bins were used for both age groups. To capture longer-lasting effects emerging over the *N*_2_ wave, 300 ms bins were used for adults and 200 ms bins were used for infants.

In adults, the rolling bins approach identified three windows of interest, each corresponding to a wave in the auditory evoked potential: 80–180 ms (*N*_1_ wave), 180–280 ms (*P*_2_ wave) and 370–670 ms (*N*_2_ wave). In infants, the rolling bins approach identified three windows of interest, two corresponding to the timing of the adult *P*_2_ wave: 150–250 ms and 225–325 ms; a third corresponding to the timing of the adult *N*_2_ window: 400–600 ms.

## 4 Results

### 4.1 Adults

#### 4.1.1 Response to expected word form

The waveform in Fig. 2(top), shows a sequence of deflections characteristic of the *PNP* complex: a small positive deflection peaking at about 50 ms (the *P*_1_ wave), followed by a large negative deflection peaking at about 100 ms (the *N*_1_ wave), and a bifidal positive deflection (the *P*_2_ wave) with a large primary peak at about 200 ms and a smaller secondary peak at about 360 ms. A long-lasting negative deflection is also evident, lasting until the end of the analysis window (the *N*_2_ wave).

The static topographic maps show no hemispheric differences during time windows capturing the *N*_1_ wave (80–180 ms) and the *P*_2_ wave (180–280 ms) indicating that the coronal orientation of the dynamic scalp bands is representative of the general pattern of effects during the *PNP* complex.

The dynamic scalp bands show that the *N*_1_ wave has a broad, slightly posterior distribution, while the primary peak of the *P*_2_ has a more frontal distribution. The dynamic scalp bands also make clear that the frontally distributed secondary peak of the *P*_2_ wave is temporally superimposed upon the posterior onset of the *N*_2_ wave, a feature difficult to interpret from the ERP waveforms or the topographic maps alone. Furthermore, the topographic map spanning the *N*_2_ period hints at a moderate hemispheric asymmetry in this time period.

#### 4.1.2 Effects of unexpected word forms

Inspection of the waveforms in Fig. 4(top) shows substantial coherence in the structure of the *PNP* complex across all three conditions. This coherence is an outcome of the fixed SOA, which ensured each auditory onset occurred at the same point in processing triggered by the visual stimulus.

Inspection of the dynamic scalp bands in Fig. 5(top) reveals that around the timing of the *N*_1_ wave, both unexpected word forms elicit more positivity over central and posterior regions, compared to expected word forms. This *N*_1_ effect overlaps with the onset of a second effect: a strong, relative negativity maximally recorded over the frontal poles during the timing of the primary *P*_2_ wave. The *P*_2_ effect also appears to be stronger and more broadly distributed in the pseudoword condition than in the mispronunciation condition. The inverse polarity, timing and distribution across coronal scalp bands of the *N*_1_ and *P*_2_ effects suggest that they are discrete effects.

After the *PNP* complex, effects elicited by mismatching conditions return to zero, followed by a period in which both mismatching conditions elicit an *N*400 effect: relative negativity during the timing of the *N*_2_ wave, maximally recorded over the right frontal regions (see Fig. 6 top).

The results of ANOVAs supporting these observations are described below. Effects and interactions of condition are summarised in Fig. 6(top) alongside static topographic maps corresponding to each time window.

*N*_1_ *effect (80–180 ms window).* The pseudoword/word comparison revealed a main effect of band (*F*(4, 72) = 5.7, *p* < . 001, $\eta_{p}^{2}=.24$) and an interaction of condition with band (*F*(4, 72) = 9.1, *p* < . 001, $\eta_{p}^{2}=.34$). The mispronunciation/word comparison revealed a main effect of condition (*F*(1, 18) = 3.8, *p* = . 07, $\eta_{p}^{2}=.17$), a main effect of band (*F*(4, 72) = 6.7, *p* < . 001, $\eta_{p}^{2}=.27$) and an interaction of condition with band (*F*(4, 72) = 9.0, *p* < . 001, $\eta_{p}^{2}=.34$). The interactions confirm that the effect of condition differs according to scalp band, with the centro-posterior *N*_1_ effect accompanied by frontal negativity from the onset of the *P*_2_ effect. Clarifying the independence of the *N*_1_ effect, repeated measures ANOVAs investigating the influence of condition and temporal region (Left: T3, T5; Right: T4, T6), revealed a main effect of condition in both the pseudoword/word comparison (*F*(1, 18) = 6.5, *p* < . 05, $\eta_{p}^{2}=.27$) and the mispronunciation/word comparison (*F*(1, 18) = 11.6, *p* < 0.01, $\eta_{p}^{2}=.39$).

*P*_2_ *effect (180–280 ms window).* The pseudoword/word comparison revealed a main effect of condition (*F*(1, 18) = 7.6, *p* < . 05, $\eta_{p}^{2}=.30$) and an interaction of condition with band (*F*(4, 72) = 7.5, *p* < . 001, $\eta_{p}^{2}=.29$). The mispronunciation/word comparison revealed a main effect of hemisphere (*F*(1, 18) = 10.2, *p* < . 01, $\eta_{p}^{2}=.36$), and an interaction of condition with band (*F*(4, 72) = 6.1, *p* < . 001, $\eta_{p}^{2}=.25$)). The interactions confirm that the influence of condition differs according to scalp band, as driven by the frontal negativity. The pseudoword/mispronunciation comparison revealed a main effect of condition (*F*(1, 18) = 8.9, *p* < . 01, $\eta_{p}^{2}=.33$), along with a main effect of band (*F*(4, 72) = 2.7, *p* < . 05, $\eta_{p}^{2}=.13$), indicating that the *P*_2_ effect is stronger in the more deviant pseudoword condition, where it is also detected over a larger scalp region.

*N*400 *effect (370–670 ms window).* The pseudoword/word comparison revealed a main effect of condition (*F*(1, 18) = 6.9, *p* < . 05, $\eta_{p}^{2}=.28$), a main effect of band (*F*(4, 72) = 6.1, *p* < . 001, $\eta_{p}^{2}=.25$), an interaction between condition and hemisphere (*F*(1, 18) = 7.3, *p* < . 05, $\eta_{p}^{2}=.29$), and an interaction between condition, band and hemisphere (*F*(3.4, 61.4) = 2.5, *p* = . 06, $\eta_{p}^{2}=.12$). The mispronunciation/word comparison revealed a main effect of condition (*F*(1, 18) = 10.6, *p* < . 01, $\eta_{p}^{2}=.37$), a main effect of band (*F*(4, 72) = 5.6, *p* = . 001, $\eta_{p}^{2}=.24$), an interaction of condition with band (*F*(4, 72) = 3.8, *p* < . 01, $\eta_{p}^{2}=.18$) and an interaction of condition with hemisphere (*F*(1, 18) = 4.2, *p* = . 055, $\eta_{p}^{2}=.19$).

#### 4.1.3 Discussion

As predicted, both unexpected word forms induced a chain of effects in the auditory evoked potentials. This chain of effects consists of both short-lasting sub-lexical effects during the *PNP* complex and longer-lasting *N*400 effects indexing semantic integration. Two discrete short-lasting effects differing in polarity and distribution are identified during the *PNP* complex: temporal/centro-parietal positivity during the *N*_1_ wave, and fronto-central negativity during the *P*_2_ wave. These effects overlap in time, as the onset of the *P*_2_ effect emerges during the *N*_1_ effect, and both manifest as a reduction in the amplitude of the underlying waveform.

Given the timing, morphology and sensitivity to degree of deviation from the expected word form, the modulation of the adult *P*_2_ wave appears to be a PMN effect elicited by deviation from the expected sequence of phonemes. Our results are in accordance with the findings by Desroches et al. (2008) and Newman and Connolly (2009), who also report a frontal negativity elicited by the phonological mismatch around the timing of the *P*_2_ wave.

This study also identified an earlier discrete effect, maximal over the temporal sites, whereby unexpected word forms generated a reduced amplitude during the *N*_1_ wave. This effect corresponds to a short-lasting effect reported by Friedrich and Friederici (2005) over a 100–250 ms time window, which they refer to as a ‘lexical priming effect’, and an apparent reduction in the amplitude of the temporal *N*_1_ wave visible in the published waveforms in Desroches et al. (2008). Effects in all three studies share polarity, and occur in contexts where expectations about upcoming word forms have been systematically manipulated. It is possible that effects could be due in part to physical characteristics of the audio stimuli, as intensity and frequency are known to modulate the *N*_1_ wave (Näätänen and Picton, 1987). However, as neither Friedrich and Friederici (2005) nor Desroches et al. (2008) make available acoustic analyses of their auditory stimuli, it is unclear whether their early effects are driven by acoustic differences or by experimental manipulations.

In the current study, acoustic differences between the different audio tokens in the word condition were greater than acoustic differences between word/mispronunciation pairs, in which each mispronunciation was closely matched to the corresponding word (see supplementary material S2 for details). If acoustic features of the stimuli were solely responsible for the *N*_1_ effect in this study, we would predict a larger *N*_1_ effect for the word/pseudoword contrast (in which acoustic characteristics of pseudowords vary substantially from the word condition) than for the word/mispronunciation contrast (in which acoustic characteristics of the stimuli are closely matched). As no experimental effects were observed between the mispronunciation condition and the pseudoword condition, it is possible that expectations about upcoming speech may have contributed to this *N*_1_ effect.

Long-lasting *N*400 effects induced by unexpected word forms over the *N*_2_ wave occurred after differences between conditions returned to zero following the early sub-lexical effects during the PNP complex, strongly suggesting that these early effects are discrete from *N*400 effects. Indeed, supplementary ANOVA analyses run over a 100 ms window between the PNP complex and the window of interest for the *N*400 effect (270–370 ms) confirm that no significance effects or interactions with condition were observed during this period. See supplementary material S4 for details.

### 4.2 Infants

#### 4.2.1 Response to expected word forms

Inspection of the grand average ERP waveforms in Fig. 3 reveals a series of deflections representing the obligatory auditory processing response in infants, overlaid on a strong negative-going slow wave, which represents the final stages of the infant visual evoked potential (longer-lasting in infants than in adults). As slow waves of this magnitude mask the polarity of higher frequency deflections of the auditory response when plotted as either dynamic scalp bands or topographic maps, these plots are omitted for infants. However, substantial concordance is observed between the adult and infant sequence of deflections, as peaks of inverse polarity occur at similar times: infants show a clear positive deflection around the timing of the adult *N*_1_, followed by a pair of negative deflections around the timing of the adult bifidal *P*_2_ wave.

#### 4.2.2 Effects of unexpected word forms

Inspection of the waveforms in Fig. 4(bottom) shows that the auditory evoked potentials for all three conditions are superimposed on a negative going slow wave which, as noted above, corresponds to activity lingering from the visual evoked potential in response to the visual stimulus. It is clear, however, that the fixed SOA between the onset of visual and auditory stimuli built into the design of the experiment ensured an equal slope for this slow wave across all conditions.

It is evident that the morphology of the early evoked potential shows substantial coherence across word and mispronunciation conditions, but is less structured in the pseudoword condition, a feature of the ERP which may have been be due to infants’ general dislike of this condition.^2^

Inspection of dynamic coronal bands in Fig. 5(bottom) reveals that infants exhibit a similar pattern of effects to adults. However, during the timing of the *PNP* complex, the sequence of effects has inverted polarity: for the infant analogue of the *N*_1_ effect, mismatching conditions generate more centro-parietal negativity in a time period corresponding to a positive deflection in the underlying waveform; for the infant analogue of the *P*_2_ effect, mismatching conditions elicit more frontal positivity in a time period corresponding to an underlying negative deflection. Like adults, these effects manifest as a reduction in the amplitude of the underlying peaks of the *PNP* complex. Following these effects, unexpected word forms induce a long-lasting negativity evident mainly over fronto-central regions, sharing polarity and topography with the adult *N*400 effect.

The results of ANOVAs clarifying these observations are described in detail below, and effects and interactions of condition are summarised in Fig. 6(bottom) alongside static topographic maps corresponding to each time window.

*P*_2_*-like effect.* During the 150–250 ms window, the pseudoword/word comparison revealed a main effect of condition (*F*(1, 17) = 4.8, *p* < . 05, $\eta_{p}^{2}=.22$) and a main effect of band (*F*(4, 68) = 26.2, *p* < . 001, $\eta_{p}^{2}=.61$). By contrast, the mispronunciation/word comparison revealed only a main effect of band (*F*(4, 68) = 39.9, *p* < . 001, $\eta_{p}^{2}=.70$). The pseudoword /mispronunciation comparison revealed a main effect of condition (*F*(1, 17) = 5.4, *p* < . 05, $\eta_{p}^{2}=.24$) and a main effect of electrode band (*F*(4, 68) = 27.0, *p* < . 001, $\eta_{p}^{2}=.61$), indicating that the influence of an unexpected word form is larger in the pseudoword condition, in this time window. During the 225–325 ms window, the pseudoword/word comparison revealed a main effect of band (*F*(4, 68) = 17.5, *p* < . 001, $\eta_{p}^{2}=.51$), and an interaction between condition and hemisphere (*F*(1, 17) = 3.1, *p* < 0.1, $\eta_{p}^{2}=.15$). The mispronunciation/word comparison revealed a main effect of condition (*F*(1, 17) = 4.8, *p* < . 05, $\eta_{p}^{2}=.22$), alongside a main effect of band (*F*(4, 68) = 19.6, *p* < . 001, $\eta_{p}^{2}=.54$) and an interaction of condition with hemisphere (*F*(1, 17) = 4.9, *p* < . 05, $\eta_{p}^{2}=.22$). The pseudoword/mispronunciation comparison revealed a main effect of condition (*F*(1, 17) = 3.6, *p* = . 07, $\eta_{p}^{2}=0.18$) and a main effect of electrode band (*F*(4, 68) = 17.4, *p* < . 001, $\eta_{p}^{2}=.51$), indicating that the influence of an unexpected word form is larger in the mispronunciation condition in this time window.

*N400-like effect.* During the 400–600 ms window, the pseudoword/word comparison revealed a main effect of band (*F*(4, 68) = 45.9, *p* < . 001, $\eta_{p}^{2}=.73$), and a three-way interaction between condition, band and hemisphere (*F*(2.9, 49.7), *p* = . 08, $\eta_{p}^{2}=.12$). This interaction, along with a two-way interaction of condition with hemisphere, was significant to the *α*-level of.05 in the majority of infants (16 out of 18). The mispronunciation /word comparison revealed no effects or interactions with condition, only a main effect of band (*F*(4, 68) = 34.6, *p* < . 001, $\eta_{p}^{2}=.67$). The topographic maps in Fig. 6(Bottom) show relative negativity in temporal and fronto-central regions, particularly in the pseudoword condition.

#### 4.2.3 Discussion

Unexpected word forms induce a chain of effects in the infant auditory evoked potential: short-lasting effects on the early auditory evoked potential followed by a period of long-lasting negativity only achieving statistical significance in the pseudoword condition.

The *P*_2_-like effect manifests as a relative positivity reaching maximum significance in different time windows following the two unexpected word form conditions. As evident in Fig. 6 (Bottom), the first window captures experimental effects induced by the pseudoword condition, which differs from the expected word condition from the very first speech segment, while the second window captures experimental effects induced by the mispronunciation condition, which differs only in the medial vowel. The relative timing of the windows capturing the *P*_2_ effects could either be due to the magnitude of the mismatch (greater mismatch in the pseudoword condition) or to the relative timing of the first speech segment which mismatches (earlier in the pseudoword condition).

The morphology of this *P*_2_-like effect is in accordance with findings from auditory-only paradigms (Mills et al., 2004, Thierry et al., 2003, familiar/unfamiliar words, 14- and 11-month-olds), and also with results from picture–word paradigms (Friedrich and Friederici, 2005, words/pseudowords, 12-month-olds). Furthermore, as is the case for the adult *P*_2_ effect, this infant effect represents a reduction in amplitude of the underlying waveform in time windows of the auditory evoked potential where peaks occur. This concordance suggests that the infant *P*_2_-like effect could be an infant analogue of the adult PMN effect.

The *N*400-like effect in the pseudoword/word contrast is in accordance with previously reported findings in infant picture–word mismatch paradigms (e.g., Friedrich and Friederici, 2005, 19-month-olds) and is also similar to the long-lasting adult *N*400 effect reported in this paper. By comparison, negativity in the mispronunciation/word contrast did not reach significance over this time window. This suggests that while infants are able to detect mispronounced vowels (as indexed by their earlier *P*_2_-like effect), it could be the case that mispronounced vowels do not constitute adult-like word-picture mismatches for 14-month-olds. That is to say, infants of this age may detect vowels which differ slightly from the expected word form, but not reject them in the context of a familiar object. Alternatively, as the mismatch in the mispronunciation condition was more subtle than in the pseudoword condition, it may be the case that the induced effect is too small to be detected given the well known, low signal-to-noise ratio in infant ERP studies.

The pattern of effects reported here differs from those in Mani et al. (2011), in which pseudowords and mispronunciations of medial vowels were also employed. In their study, a sustained period of relative negativity was evident from 200 ms, until the end of the trial. In the current study, the *P*_2_-like effect was evident as relative positivity for unexpected word forms, followed by an *N*400-like negativity detected over a later, discrete, time window. The effects have opposite polarity and each is shorter than the period of negativity evident in Mani et al. (2011). Differences between their findings and the current study are best understood in the light of the following differences in experimental approach: (a) the fixed SOA in this study reduced inter-trial differences in the residual visual evoked potential and (b) the attenuation of slow-waves allowed greater clarity in the structure of short-lasting effects. In this context, it is possible that the long-lasting negativity reported by Mani et al. (2011) (beginning early and continuing over the *N*400 region), may well overlay other short-lasting early effects. As the infant chain of effects in the current study is in concordance with the adult pattern of results, we are confident that the infant effects reported here reliably reflect detection of unexpected word forms.

This infant chain of effects shares similarities with a number of infant studies, as well as concordance with adult studies, if the early effects are acknowledged as reductions in the amplitude of the underlying waveform in response to unexpected word forms. This suggests 14-month-olds share with adults some underlying processing in their detection of unexpected word forms, an effect which is clearest when the deviations from expectation are large and start early, as is the case in the pseudoword condition.

## 5 Conclusions

In a context where pictures provide expectations about upcoming speech, unexpected word forms elicit a chain of effects in both adults and 14-month-old infants: early sub-lexical effects occurring during the *PNP* complex, followed by a discrete semantic integration effect occurring over the *N*_2_ wave. These results indicate that for infants, like adults, the neural correlates of detection of subtle deviations from expected word forms are evident early in the auditory evoked potential.

Sub-lexical *P*_2_ effects were reliable in both adults and infants, for both types of unexpected word form. To the best of our knowledge, this is the first time that adult sensitivity to minimal deviations in word-medial vowels has been demonstrated in an ERP paradigm where pictures provide context. While *P*_2_-like effects with the same amplitude modulation have previously been reported in infancy, they have not previously been reported in response to such small deviations from the expected word form.

This study also identified an earlier, short-lasting effect in adults, the *N*_1_ effect, starting less than 100 ms after auditory onset, and spanning the *N*_1_ wave. This effect provides evidence for high speed incremental processing of speech in a scenario in which visual information may provide expectations about upcoming speech.

The *N*400 effect was reliable for both types of unexpected word form in the adult sample, and in response to completely novel pseudowords in the infant sample.

Comparison of the infant and adult responses permits identification of the infant effects as analogues to the adult chain of effects, thereby providing evidence for shared mechanisms over development. The dynamic scalp bands introduced in this paper allowed informative visualisation of similarities in the chain of effects observed across age groups, despite the groups’ underlying waveforms having different structures. By bringing together spatial and temporal information in a single plot, this visualisation method highlights the tight concordances between adult and infant ERP effects generated by linguistic stimuli presented in a visual context.

## Conflict of interest

The authors have no conflict of interest.

## Footnotes

1 Results from Condition × Region (frontal/centro-posterior) ANOVAs for electrodes in the midline region are omitted for brevity, as they agree with the results of the main analysis.

2 A number of infants exhibited behaviour suggesting annoyance in response to pseudoword trials, including frowning, head-shaking, turning away from the screen, or correcting the speaker (e.g., ‘No! Dog!’). While such unattended trials or trials containing artifacts due to gross muscle movements were excluded, analysed trials may contain markers of similar negative affect states, potentially influencing the morphology of the auditory evoked potential in this condition.
