# Supplementary material for: ERP correlates of unexpected word forms in a picture–word study of infants and adults
Source: Dev Cogn Neurosci. 2012 Jan 16;2(2):223–34. doi: 10.1016/j.dcn.2012.01.003 (PMC3336206; doi:10.1016/j.dcn.2012.01.003)
Supplement: Supplementary file 1 [file mmc1.pdf]

Supplementary material

S1. List of visual and auditory stimuli

Word (W) - Mispronunciation (M) Pairs

Visual stimulus		Audio token	Vowel height	Vowel backness	Dur. (ms)	Comp. %[a]
**bib**	W	/bɪb/	High	COR	490	53.2
	M	/bɛb/	Mid	COR	494	
**fish**	W	/fɪʃ/	High	COR	790	45.9
	M	/fɛʃ/	Mid	COR	701	
**fridge**	W	/frɪdʒ/	High	COR	870	42.3
	M	/frɛdʒ/	Mid	COR	794	
**bread**	W	/brɛd/	Mid	COR	721	55.0
	M	/brɪd/	High	COR	602	
**hand**	W	/hænd/	Low	COR	709	53.2
	M	/hɛnd/	Mid	COR	788	
**hat**	W	/hæt/	Low	COR	778	56.8
	M	/hɛt/	Mid	COR	565	
**foot**	W	/fʊt	High	DOR	806	66.7
	M	/fɒt/	Mid	DOR	740	
**dog**	W	/dɒg/	Mid	DOR	574	89.2
	M	/dʊg/	High	DOR	585	
**sock**	W	/sɒk/	Mid	DOR	756	72.1
	M	/sʊk/	High	DOR	706	
**doll**	W	/dɒl/	Mid	DOR	743	55.0
	M	/dʊl	High	DOR	598	
**block**	W	/blɒk	Mid	DOR	721	45.0
	M	/blʊk/	High	DOR	521	
**bottle**	W	/bɒt.l/	Mid	DOR	726	76.6
	M	/bʊt.l/	High	DOR	616	

a. Comprehension rates according to Dale and Fenson (1996) at fourteen months-of-age

Pseudowords

Audio token	Vowel height	Vowel backness	Dur. (ms)
/frɪb/	High	COR	733
/bɛf/	Mid	COR	446
/blɛv/	Mid	COR	629
/fɛp/	Mid	COR	640
/fæsk/	Low	COR	795
/sæb/	Low	COR	724
/suːb/	High	DOR	783
/dɒm/	Mid	DOR	575
/fɒtʃ/	Mid	DOR	798
/hɔːrb/	Mid	DOR	740
/sɒθ/	Mid	DOR	692
/dʊlv/	Low	DOR	753

S2. Stimulus Pitch[b]

b. F_0 tracks calculated using Praat Version 5.2.35 (Boersma & Weenink, 2011, *Praat: doing phonetics by computer.* http://www.praat.org/), Pitch range: 75-500Hz, autocorrelation method, manually corrected for creaky phonation prior to voiceless stop consonants.

S3. Soothers, Engagers and Eye-catchers (SEE)

Twenty of the SEE cartoons lasted 2s, and were designed to reward attention, or attract eye-gaze. They employed moving or looming shapes (e.g., stars, rectangles, circles, flowers), accompanied by real-world sound effects from instruments, toys, and human sources (e.g., popping a thumb across the mouth). Five of the SEE cartoons lasted 3-5s and were more structured, with slower motion and a more obvious narrative (e.g., leaves falling), designed to maintain calm, focussed attention. Five of the SEE cartoons were longer, lasting 8-10s, and were designed to be soothing, with a repetitive structure (e.g., a musical scale). SEE cartoons were triggered using three separate buttons on the gamepad, allowing the pace of each session to be tailored to the infant's changing attentional state. As these cartoons were more variable than the experimental stimulus set, they provided additional motivation for infants to keep watching the screen for low frequency events. For an example, see startjumps.mov uploaded with submission.

S4. Supplementary analyses

Adult window between P_2 and $N400$ windows of interest

270-370ms window Neither pseudoword/word comparison nor mispronunciation/word comparison revealed any significant main effects or interactions with condition. The pseudoword/word comparison: main effect of condition ($F(1, 18) = .005$, $p = .94$, $\eta_p^2 < .001$), interaction between condition and scalp band ($F(4, 72) = .40$, $p = .81$, $\eta_p^2 = .02$), interaction between condition and hemisphere ($F(1, 18) = .49$, $p = .49$, $\eta_p^2 = .03$). The mispronunciation/word comparison: main effect of condition ($F(1, 18) = .001$, $p = .99$, $\eta_p^2 < .001$), interaction between condition and scalp band ($F(4, 72) = .70$, $p = .59$, $\eta_p^2 = .04$), interaction between condition and hemisphere ($F(1, 18) = .05$, $p = .82$, $\eta_p^2 < .01$).

Figure S2a. F_0 tracks for words (solid black line) and mispronunciations (dashed black line) showing similarity between paired audio tokens where only the vowel differed in height.

Figure S2b. F_0 tracks showing spread of pitch within each condition. TOP words, MIDDLE mispronunciations, BOTTOM pseudowords.

Figure S2a

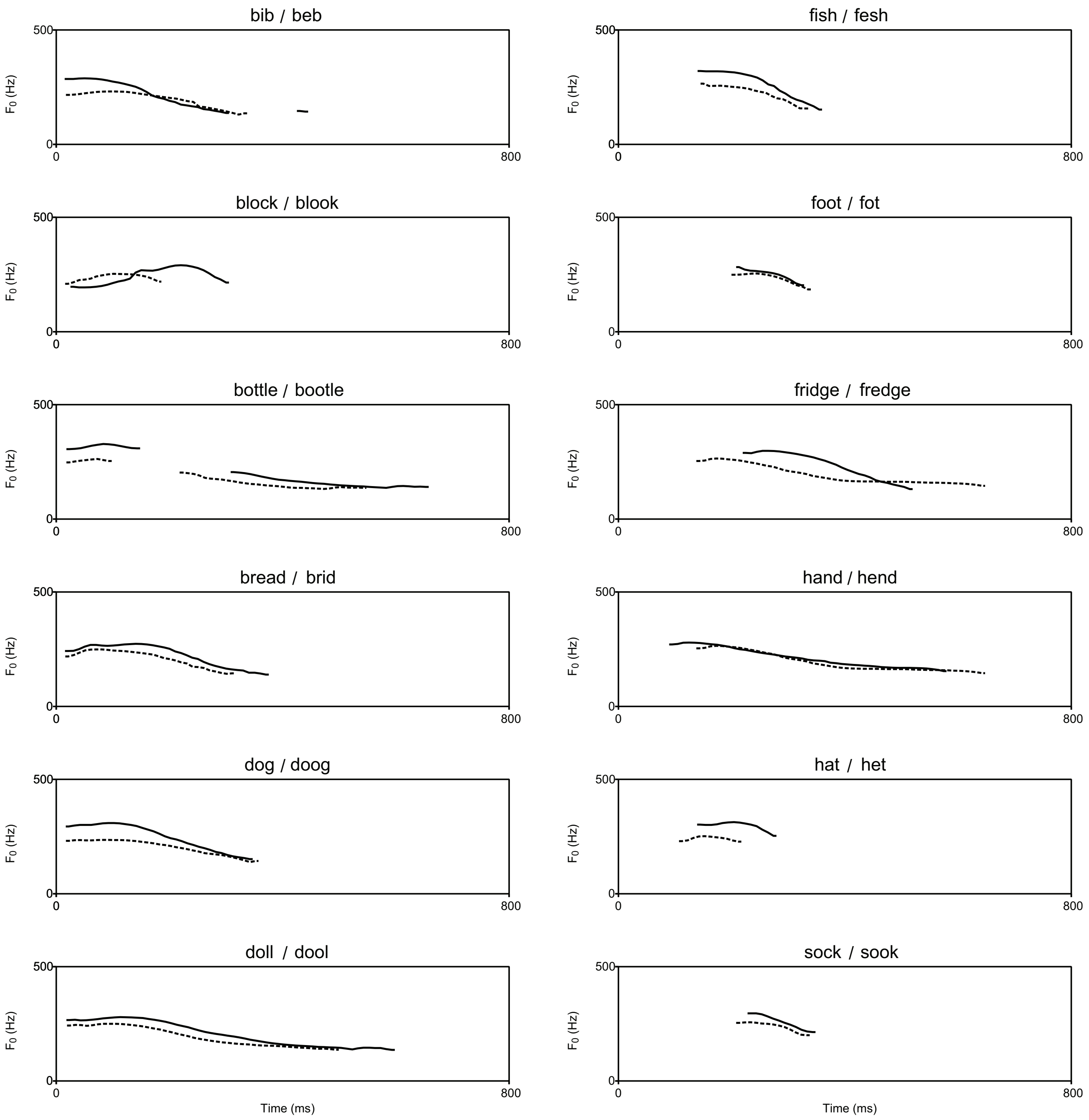

bib / beb
fish / fesh
block / blook
foot / fot
bottle / bootle
fridge / fredge
bread / brid
hand / hend
dog / doog
hat / het
doll / dool
sock / sook
F0 (Hz)
500
0
800
Time (ms)

Figure S2b

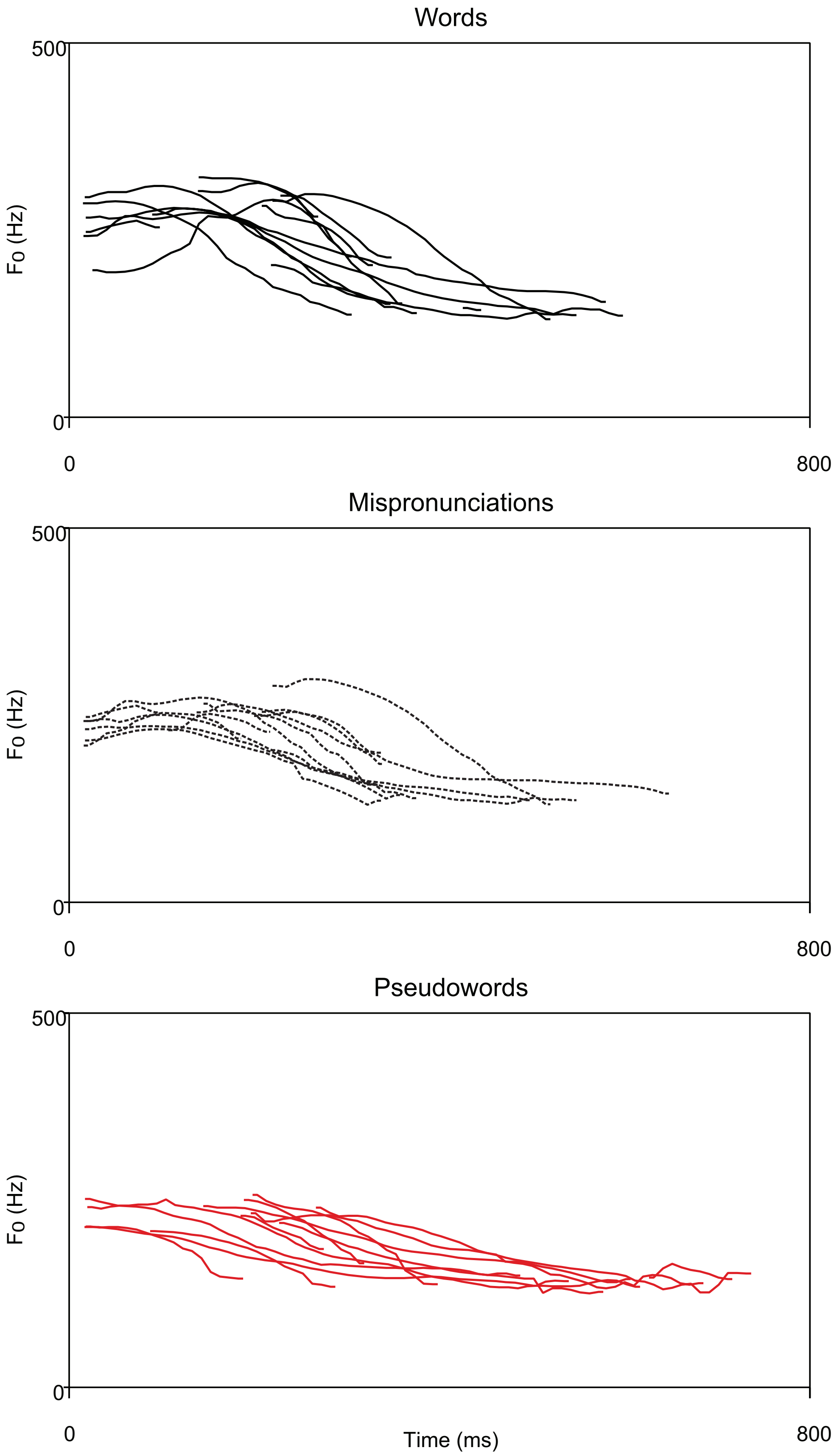
Words
500
0
F0 (Hz)
0
800
Mispronunciations
500
0
F0 (Hz)
0
800
Pseudowords
500
0
F0 (Hz)
0
800
Time (ms)
